# A Rapidly Growing Cardiac Mass—Malignant or Benign?

**DOI:** 10.1016/j.case.2023.04.004

**Published:** 2023-05-23

**Authors:** Amit Rout, Mounica Vorla, Afolasayo A. Aromiwura, Siddharth V. Pahwa, Marcus F. Stoddard, Mark S. Slaughter, Dinesh K. Kalra

**Affiliations:** a Division of Cardiology, University of Louisville, Louisville, Kentucky; b Department of Medicine, University of Louisville, Louisville, Kentucky; c Department of Cardiothoracic Surgery, University of Louisville, Louisville, Kentucky

**Keywords:** Cardiac tumors, Atrial myxoma, Pulmonary hypertension, Echocardiography

## Abstract

• Rapidly growing cardiac tumors can be benign or malignant.
• Rarely, cardiac myxomas may grow rapidly, causing heart failure or obstructive symptoms.
• Echocardiography remains the first-line imaging for cardiac tumors.
• Multimodality imaging with CCT, CMR, and PET may aid in diagnosis.

## Graphical abstract

**Figure:**
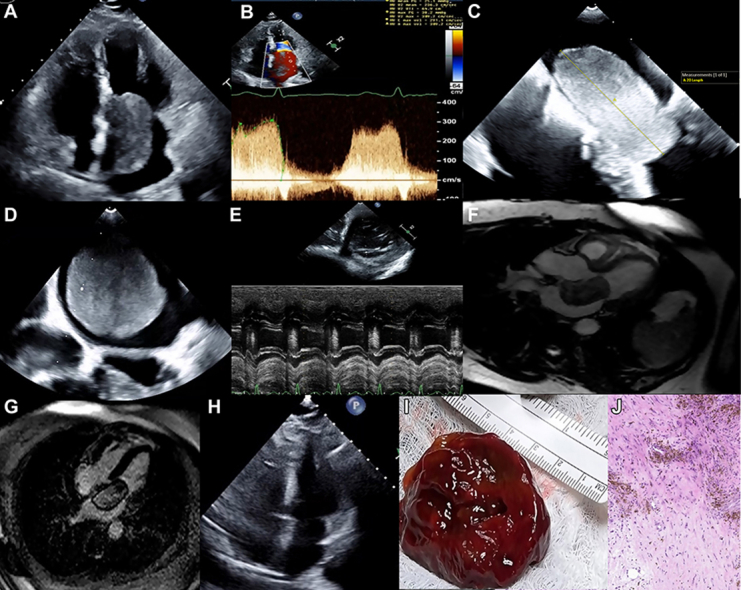

## Introduction

Primary cardiac tumors occur infrequently, with an incidence of 0.0017% to 0.19% according to an autopsy series performed in nonselected populations.^1^^,^^2^ Secondary cardiac tumors (usually metastases to the heart) outnumber primary cardiac tumors 20 to 1. Cardiac myxomas, often called the “great masquerader,” are the most common among them, with an annual incidence of 0.5 to 1 case per million.^3^ Signs and symptoms of myxomas usually depend on their size, mobility, and location. The triad of embolism, intracardiac obstruction, and constitutional symptoms is the classic clinical manifestation of cardiac myxoma.^4^ Transthoracic (TTE) and transesophageal (TEE) echocardiography is often the first-line imaging technique for the diagnosis of cardiac masses, with TEE yielding a higher sensitivity of 100% versus 95%.^5^ Other modalities such as cardiac computed tomography (CCT) or cardiovascular magnetic resonance (CMR) are used if diagnostic confusion persists after TTE or TEE as they provide superior anatomical delineation of cardiac masses.

Here we describe a rare case of a rapidly growing left atrial (LA) myxoma presenting as heart failure and the role of multimodality cardiac imaging in confirming the diagnosis and planning the preoperative management.

## Case Presentation

**Figure 1:**
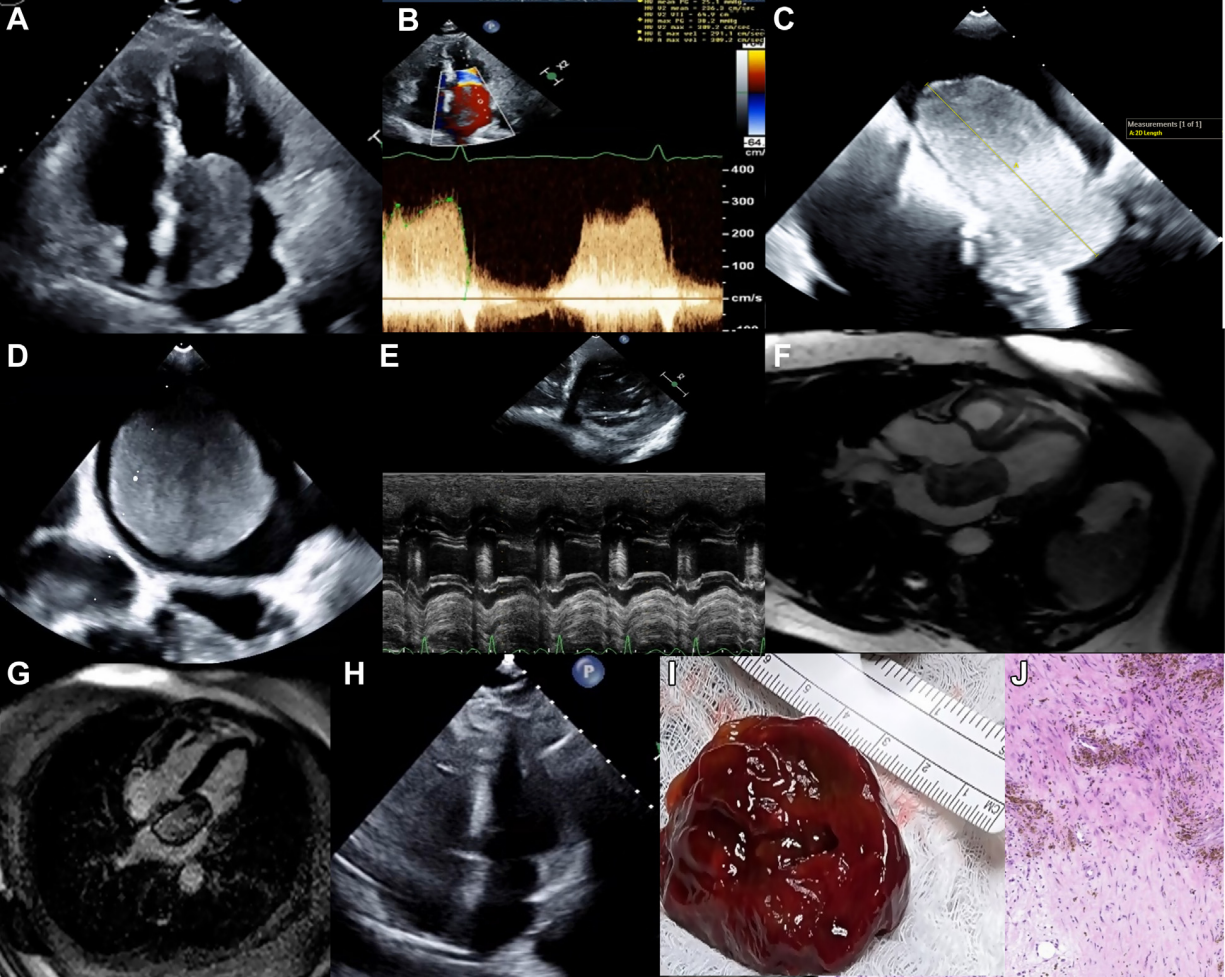
Two-dimensional TTE apical 4-chamber view **(A)**, continuous-wave Doppler of the mitral valve **(B)**; two-dimensional TEE at the midesophageal level (0°) **(C)**, transgastric level (64°) **(D)**, and M-mode **(E)**; CMR axial 3-chamber display, steady-state free precession, diastolic phase (**F**), and LGE sequence in an axial 4-chamber display **(G)**; two-dimensional TTE apical 4-chamber view 9 months prior to presentation **(H)**, intraoperative image of excised mass **(I)**, and histopathology showing lepidic cells in a myxoid stroma **(J)**.

A 51-year-old woman presented with progressive shortness of breath and lower extremity edema for 1 week. Physical exam revealed pulmonary congestion, elevated jugular venous pressure, 2+ leg edema, a grade 2/6 diastolic murmur, and an early diastolic “plop” at the apex. Transthoracic echocardiography showed a large LA mass with diastolic prolapse into the left ventricle (LV) causing severe mitral stenosis (mean gradient, 25 mm Hg; Figure 1A and B). Left ventricular (LV) ejection fraction was 58%, and pulmonary artery systolic pressure was 62 mm Hg. Transesophageal echocardiography showed a 6.0 × 5.8 × 2.9 cm encapsulated mass that occupied almost the entire left atrium (LA; Figure 1C and D, Videos 1 and 2). M-mode and color flow Doppler showed significant LV inflow obstruction (Figure 1E). The mass was attached to the atrial septum at the fossa ovalis by a short stalk. Cardiac magnetic resonance imaging showed that the mass was isointense on T1-weighted images with no fat suppression and hyperintense on T2-weighted images and had extensive, heterogenous late gadolinium enhancement (LGE; Figure 1F and G, Videos 3 and 4). A TTE performed 9 months prior showed no mass, indicating a very rapid growth rate of ∼6 mm/month (Figure 1H, Videos 5-7). The patient underwent complete surgical excision of the mass, which was soft and gelatinous appearing with scattered hemorrhages. Histopathology showed characteristic lepidic, polygonal cells in a loose, myxoid stroma confirming the diagnosis of myxoma (Figure 1I and J, Video 8). Thirteen months after surgery, the patient is asymptomatic and doing well.

## Discussion

There are very rare case reports of rapidly growing LA myxomas, however, not to this size in this short a span of time or rate of growth (6 mm/month; the average rate of growth is reported to be 1 mm/month), and none with this degree of LA encroachment and severe mitral stenosis producing rapid heart failure symptoms.^1^ Additionally, the absence of any systemic symptoms such as fever, abnormal inflammatory biomarkers (interleukin-6, C-reactive protein), or embolic phenomena with such a large myxoma was unusual. Even though the very rapid rate of growth suggested a malignant neoplasm, other features argued against malignancy, namely, its location in the LA as a solitary lesion with origin in the fossa ovalis, lack of penetration through tissue planes, absence of invasion of the great vessels, myocardium, and pericardium, and no primary neoplasm elsewhere (e.g., in the lungs, kidney, or breast).^6^ Table 1 lists features that can help differentiate between benign and malignant cardiac masses. Our patient had no family history of tumors and had a solitary myxoma with no other features of the Carney complex, lentigines, atrial myxomas, and blue nevi, or nevi, atrial myxoma, myxoid neurofibromas, and ephelides syndromes.

**Table 1:** Clinical and imaging features that help distinguish between benign and malignant cardiac masses

Feature	Favors benign cardiac mass	Favors malignant cardiac mass
Size and rate of growth	Smaller in size when diagnosed; slow growing (1-4 mm/month)	Usually larger in size; faster growing (5-15 mm/month)
Location	LA location	Right atrial location
Systemic features	Usually absent (except fever in 14% of myxomas)	Weight loss; constitutional symptoms and symptoms of primary malignancy
Pericardial involvement	Unusual	Pericardial effusion commonly seen, usually hemorrhagic; pericardial invasion and/or seeding may also be present (nodular studding)
Myocardial infiltration	Usually no involvement (except fibroma or rhabdomyoma or lipoma, which may arise in the myocardium)	Direct invasion of myocardial tissue from outside the heart or from a cardiac chamber cavity often seen
Coronary artery involvement	No involvement, although extrinsic compression can occur with large benign masses (fibrotic encasement of the coronary arteries may occur in Erdheim-Chester disease)	Coronary arterial encasement may be seen in lymphoma; other malignant tumors may directly invade coronaries
Valvular dysfunction	Usually not (but papillary fibroelastomas usually involve the valve leaflets and if large may cause regurgitation); carcinoid tumors can cause right-sided valvular thickening	Direct invasion of valve apparatus or annulus is not common but can occur
Arrhythmia	Less common with benign masses unless large in size or strategic location (e.g., neuroendocrine tumors may involve the area near the atrioventricular node)	Atrioventricular block, bundle branch block, conduction abnormalities, and ventricular arrhythmias more common with myocardial and epicardial invasion by malignant masses
Systemic or pulmonary embolism	May occur with small or large tumors depending on the chamber or valvular location (e.g., valve papillary fibroelastoma, myxoma)	Commonly seen in metastatic tumors; may also occur from thrombus associated with the tumor
Extracardiac masses	Absent (except syndromes such as lentigines, atrial myxomas, and blue nevi and nevi, atrial myxoma, myxoid neurofibromas, and ephelides may have extracardiac myxomas, neurofibromas, and other tumors)	Usually present (site of primary malignancy, or other metastatic sites)
Obstruction	Valvular or outflow tract obstruction can rarely occur with large mural benign tumors, e.g., myxomas	Superior vena caval obstruction is more common with right-sided malignant lesions, e.g., angiosarcoma, metastatic lung cancer, breast cancer, lymphoma, thymoma, and germ cell tumors
LGE on CMR	No LGE (except fibromas and in most myxomas)	LGE is almost always present.
First-pass perfusion on CMR (gadolinium) or echocardiography (ultrasound-enhancing agent)	Most benign masses do not show high vascularity except for hemangiomas (“sunray” appearance).	Strong contrast uptake (high vascularity in malignant masses)

Multimodality cardiac imaging assists in tumor characterization and plan preoperative management. Aside from providing a comprehensive assessment of cardiac function and structure, CMR is uniquely suited to provide noninvasive tissue characterization of masses. Cardiac myxomas frequently have high signal intensity on T2 imaging due to their high water content (myxoid elements) and display heterogenous high signal on LGE sequences. Occasionally, thrombi on the surface of the myxoma can produce a low signal intensity on the exterior of the mass.^7^ Parametric mapping can help differentiate between the various components of a mass such as fat, iron, lipid, melanin, blood, fluid, and fibrous tissue. Cardiac computed tomography is the imaging modality of choice for the detection of calcification and can provide complementary information to CMR regarding tissue composition (e.g., cystic elements, fat, hematoma or fresh thrombus, abscess, etc.) and aid in preoperative planning, but it exposes the patient to radiation.^8^ Positron emission tomography (PET) can detect tumors (which are usually hypermetabolic), inflammation, and infection, and thus it can be useful to detect infections on implanted devices, endocarditis, or inflammatory conditions (sarcoidosis, vasculitis). Despite advances in various cardiac imaging modalities, echocardiography remains the first line in the evaluation of cardiac masses. Echocardiography has the advantage of having excellent temporal resolution (10 ms compared to 25 ms for CMR and 75-125 ms for CCT) and is preferred for small, mobile structures (such as vegetations or papillary fibroelastomas), which may be missed by CMR or CCT. Additional advantages are that it is portable and widely available.

Left-sided cardiac myxomas are managed with surgical excision, with an operative mortality of under 0.5% and a risk of recurrence of 2% to 5%.

## Conclusion

One should not judge a cardiac mass solely based on its growth rate but apply a comprehensive approach incorporating all the clinical and imaging information available. This increases the predictive accuracy for noninvasive characterization of cardiac masses. Nonetheless, histology remains the gold standard.

## Consent Statement

Complete written informed consent was obtained from the patient (or appropriate parent, guardian, or power of attorney) for the publication of this study and accompanying images.

## Ethics Statement

The authors declare that the work described has been carried out in accordance with The Code of Ethics of the World Medical Association (Declaration of Helsinki) for experiments involving humans.

## Funding Statement

The authors declare that this report did not receive any specific grant from funding agencies in the public, commercial, or not-for-profit sectors.

## Disclosure Statement

The authors report no conflict of interest.

## Supplementary Data

Video 1 Two-dimensional TEE, midesophageal zoomed 4-chamber view (0°) with color flow Doppler demonstrates the large LA mass obstructing the mitral valve outflow.

Video 2 Two-dimensional TEE, transgastric short-axis (0°) view at the level of mitral valve demonstrates the large LA mass completely obstructing the mitral valve.

Video 3 Cardiovascular magnetic resonance steady-state free precession sagittal short-axis display at the level of the aortic valve demonstrates the large LA mass.

Video 4 Cardiovascular magnetic resonance steady-state free precession 3-chamber display demonstrates the LA mass attached to the interatrial septum, passing through and obstructing the mitral valve.

Video 5 Two-dimensional TTE apical 3-chamber view with color flow Doppler, obtained 9 months prior to this presentation, demonstrates no evidence of LA mass.

Video 6 Two-dimensional TTE apical 4-chamber view with color flow Doppler, obtained 9 months prior to this presentation, demonstrates no evidence of LA mass.

Video 7 Two-dimensional TTE parasternal long-axis view with color flow Doppler, obtained 9 months prior to this presentation, demonstrates no evidence of LA mass.

Video 8 Intraoperative two-dimensional TEE, midesophageal 3-chamber view (130°) with color flow Doppler, obtained after LA myxoma excision.
